# The millipede genera *Amblyiulus* Silvestri, 1896 and *Syrioiulus* Verhoeff, 1914 in the Caucasus, with notes on their distributions (Diplopoda, Julida, Julidae)

**DOI:** 10.3897/zookeys.1048.68454

**Published:** 2021-07-13

**Authors:** Aleksandr P. Evsyukov, Sergei I. Golovatch, Dragan Ž. Antić

**Affiliations:** 1 Don State Technical University, Department of Biology and General Pathology, Rostov-on-Don, Russia Don State Technical University Rostov-on-Don Russia; 2 Institute for Problems of Ecology and Evolution, Russian Academy of Sciences, Moscow, Russia Institute for Problems of Ecology and Evolution, Russian Academy of Sciences Moscow Russia; 3 University of Belgrade, Faculty of Biology, Institute of Zoology, Belgrade, Serbia University of Belgrade Belgrade Serbia

**Keywords:** Faunistic records, key, map, new species, Pachyiulinae, Pachyiulini, taxonomy

## Abstract

In the Caucasus, the genera *Amblyiulus* Silvestri, 1896 and *Syrioiulus* Verhoeff, 1914 are shown to include two and four species, respectively: *Amblyiulus georgicus* Lohmander, 1932, from Georgia and Armenia, *A. hirtus***sp. nov.**, from Azerbaijan and Dagestan, Russia, *Syrioiulus adsharicus* (Lohmander, 1936), from Georgia, *S. continentalis* (Attems, 1903), from Azerbaijan and Iran, *S. taliscius* (Attems, 1927), from Azerbaijan, and *S. armeniacus***sp. nov.**, from Armenia. All these six species are described, illustrated, and keyed, and their distributions are mapped and discussed, based on the literature data and abundant new samples.

## Introduction

The very large family Julidae, of the basically Holarctic order Julida dominates the millipede faunas of Europe and the Mediterranean, marginally extending into the Oriental realm as well (Enghoff et al. 2015; Kime and Enghoff 2017). The subfamily Pachyiulinae, often referred to as the tribe Pachyiulini, encompasses between 15 and 20 genera, or 16–22 genera or subgenera, according to Antić et al. (2018) and Vagalinski (2020), respectively, and is characterised by the anterior gonopods being devoid of flagella, with a distinct sternum, both being fused mediobasally, and the posterior gonopods showing a mesomeral process, if any, only as an anterior branch of the opisthomere (Attems 1940; Tabacaru 1978). This group is monophyletic (Enghoff et al. 2013), temperate trans-Palaearctic, mostly restricted to the Mediterranean and ranges from Macaronesia in the west, through the entire Mediterranean, Central Asia, and central China, to Japan in the east. In the Caucasus proper, including the near-Caspian part of the Republic of Azerbaijan, but excluding the one in Iran (= both parts forming the Hyrcanian biogeographic province), this subfamily/tribe is currently known to be represented by three genera only.

The genus *Pachyiulus* Berlese, 1883 contains ca. 15 species of mostly very large julids which are largely confined to Southern and Southeastern Europe, the Near East, and the Caucasus (Enghoff et al. 2015). The Caucasus actually supports a single native species, *P. krivolutskyi* Golovatch, 1977, recently revised (Evsyukov 2016) and endemic to the western Caucasus (= Colchidan biogeographic province) within both Georgia and Russia (Kokhia and Golovatch 2020). One more congener, the eastern Mediterranean and synanthropic *P. flavipes* (C.L. Koch, 1847), has been introduced to the western Caucasus (Lohmander 1936), also being a very common, “tramp” species across Crimea (e.g., Golovatch 2008; albeit perhaps erroneously referred to as *P. varius* (Fabricius, 1781)).

The remaining two known genera of this tribe/subfamily which inhabit the Caucasus are *Amblyiulus* Silvestri, 1896 and *Syrioiulus* Verhoeff, 1914. The diagnoses and species compositions of these two genera, the main focus of the present contribution, remained unclear and confused for a long time, sometimes the latter genus being treated even as a synonym of the former (Tabacaru 1978, 1995). Mauriès (1982), in contrast to Tabacaru (1978), elevated *Syrioiulus* to a full genus and defined it primarily through a deeply bipartite posterior gonopod. He considered *Syrioiulus*, together with the monotypic *Promeritoconus* Verhoeff, 1943, from Turkey, and the species-rich genus *Amblyiulus*, as a single eastern Mediterranean lineage in the subfamily Pachyiulinae that is distinguished by the presence of eyes and, with a few exceptions only, 1+1 frontal setae, the development of apicoventral lobes on the male mandibular stipites, and of subequally high posterior gonopodal mesomeral process and opisthomere. Most, but not all, of the species from the Levant, Sporades (Greece), Caucasus, Iran and even Japan were thereby formally transferred to *Syrioiulus*. Some others from the same regions remained in or newly reassigned to *Amblyiulus*.

Enghoff (1992), in his review of *Dolichoiulus* Verhoeff, 1900, mentioned *Syrioiulus* only in passing. He, in his own outline of the pachyiuline generic classification, rediagnosed *Amblyiulus* and put the main emphasis on the structure of the posterior gonopods which show three apical processes, not two as is characteristic of several other genera, including *Syrioiulus*. Furthermore, he clearly illustrated the gonopods of *A. barroisi* (Porat, 1893), the type species of *Amblyiulus*.

The situation has become fully clarified only very recently, when first Golovatch (2018) and then Vagalinski (2020) confirmed the distinctions between *Amblyiulus* and *Syrioiulus* as lying solely in posterior gonopod conformation, also refining their diagnoses and scopes. Both these genera appear to be very similar, but differ clearly in the structure of the posterior gonopods: each of these being strongly divided into two branches (“bipartite”), i.e., a frontal (= mesomeral) and a caudal (= opisthomere) branch in *Syrioiulus* spp., vs. “tripartite”, with a third branch, a flagelliform rod, in *Amblyiulus* spp. (Golovatch 2018). A further refined account of the main differences between both these genera compared is given below.

Considering the above distinctions, *Amblyiulus* in the Caucasus appears to comprise only one described species: *A. georgicus* Lohmander, 1932. In addition, both Bababekova (1969, 1996) and Rakhmanov (1972) listed in Azerbaijan a dubious species, *A. faliocius* Attems, likely a misspelling of *taliscius*. The genus *Syroiulus* in the Caucasus presently includes three old species, all valid: *S. adsharicus* (Lohmander, 1936), *S. continentalis* (Attems, 1903), and *S. taliscius* (Attems, 1927). Vagalinski (2020), in his review and a provisional checklist of *Syrioiulus* species, emphasised the still-poor distinctions of *Syrioiulus* as opposed to a few pachyiuline genera other than *Amblyiulus*.

## Materials and methods

All material has been shared between the collections of the Zoological Museum of the Moscow State University, Russia (**ZMUM**), the Senckenberg Museum of Natural History in Görlitz, Germany (**SMNG**), and the Institute of Zoology, University of Belgrade, Serbia (**IZB**). The specimens are stored in 70% ethanol. Some parts of males (antennae, gonopods, legs, etc.) and females (vulvae and leg pairs 2) were dissected and mounted in glycerol on temporary microscopic slides. Photographs were taken using a Zeiss StereoDiscovery V.20 microscope and processed with Zeiss ZEN software. Line drawings were executed using a camera lucida attached to a Radical light-transmission microscope. Scanning electron micrographs were taken with a Zeiss CrossBeam 340 (Rostov-on-Don State Technical University, Rostov-on-Don, Russia) or a JEOL JSM-6510LV (**SMNG**) scanning electron microscope (**SEM**). For some SEM micrographs, the gonopods were glued to a small sticky plastic triangle, placed on an SEM-stub, air dried for two days in a glass filled with Silica gel and finally coated with gold. After examination, material was removed from stubs and returned to alcohol. Live animals were photographed in situ using a Canon PowerShot SX120 IS digital camera.

The distribution map was created using Google Earth Pro 7.3.3 and processed in Adobe Photoshop CS6.

A “body ring formula” indicates the number of podous (including the gonopod-bearing segment/ring) and apodous segments/rings in an individual. This formula is p+a+T where p is the number of podous body rings, a the number of apodous body rings, and T represents the telson (Enghoff et al. 1993). Only adults have been analysed in the present study. For morphological descriptions, we largely used the terminology from Minelli (2015), for descriptions of the gonopods, that of Enghoff (1992) with changes in Vagalinski (2020).

The biogeographic regionalisation of the Caucasus follows the botanical one by Menitsky (1991).

No type material of the previously described species has been revised (mostly stored in the Zoological Institute, Russian Academy of Sciences, St. Petersburg) because of the 2020–2021 COVID pandemic, and the descriptive accounts and illustrations available in the literature are sufficiently complete and clear to allow a safe species identification. Colouration is largely described from preserved material. In the catalogue sections, D stands for a description or descriptive notes, R for new or repeated records, while M is a mere mention.

### Refined characteristics of *Amblyiulus* vs. *Syrioiulus*

As shown below in the descriptions of individual species, the use of SEM allows for the distinctions between both genera concerned to be further refined. It is the opisthomere, not the entire posterior gonopod, that is bifid in *Syrioiulus*: a solenomere (with a distinct fovea or a deep saddle-like structure on top) and an anterior process (an anterior, lamellar branch adjacent to the solenomere) (Figs 7D, 10D, 12D–F, 14D). Using standard light microscopy, this anterior process often remains unnoticed, since it is very tightly appressed to the solenomere. In contrast, a third, anteromesal or lateral process/rod of the opisthomere is characteristic of *Amblyiulus* (Figs 3D, F, 5A, D–F, I, J).

As a result, it is only the structure of the opisthomere of the posterior gonopods that allows for the genera *Amblyiulus* and *Syrioiulus* to be more or less confidently diagnosed and separated. At the same time, species of *Syrioiulus* are mostly very similar in gonopodal conformation (Figs 7A–C, E, F, 10A–C, E, F, 12A–C, I, J, 14A–C, E–G), but they differ well in somatic characteristics, such as the presence or absence of eyes, frontal setae, and hairs on the rings (see also Key below). Similarly, within the genus *Pachyiulus*, several species were synonymised based solely on shared gonopodal characters (Mauriès et al. 1997), but later some have been revalidated on the basis of somatic features such as colouration (Frederiksen et al. 2012).

## Taxonomic part

### Genus Amblyiulus

Taxon classificationAnimaliaJulidaJulidae

Silvestri, 1896

9B65CB30-B6B8-5DFB-BE92-06280BF3A26A

#### Type species.

*Julus barroisi* Porat, 1893, by original designation.

#### Diagnostic remarks.

Here we follow Tabacaru’s (1978) opinion that the genera of the subfamily/tribe Pachyiulinae/-ini are best to be diagnosed using a complex of characters, both gonopodal and somatic. In the latest review of this tribe (Mauriès 1982), all genera are divided into three groups depending on the structure of the apical part of the opisthomere, viz., the presence/absence of a fovea and the presence/absence of a pseudoflagellum. He mistakenly assigned the genus *Amblyiulus* to group 3 (along with many other genera like *Dolichoiulus*), which have neither a fovea nor a pseudoflagellum. However, in accordance with our and earlier descriptions (e.g., Golovatch 2018), *Amblyiulus* has a fovea, however small, on the top of the solenomere. By the presence of a fovea and the absence of a pseudoflagellum, the genera *Parapachyiulus* Golovatch, 1979 and *Dangaraiulus* Golovatch, 1979 also join this group (Golovatch 1979, 2018). According to a number of other characters, such as the presence of frontal setae, apicoventral lobes on the male mandibles, and the mesomeral process being as high as the opisthomere, *Amblyiulus* belongs to Mauriès’ subgroup 3aa, together with the genera *Syrioiulus* and *Promeritoconus*. However, it seems noteworthy that sometimes frontal setae can be absent, while male mandibular stipites can remain unmodified.

The promere in *Amblyiulus* is narrowed in the basal third, in contrast to that in *Promeritoconus*, which is narrowed apically; in the apical part it may have one or two denticles, but sometimes none. The head can be with or without frontal setae. The eyes are mostly absent. The opisthomere of the posterior gonopod is tripartite: a solenomere, an anterior process, and an anteromesal or lateral rod, vs. bipartite in *Syrioiulus*.

#### Species included.

*Amblyiulus barroisi* (Porat, 1893), *Amblyiulus cedrophilus* (Attems, 1910), *Amblyiulus festae* (Silvestri, 1895), *Amblyiulus georgicus* Lohmander, 1932, *Amblyiulus hirtus* sp. nov., and possibly several others, but their identity requires verification (Golovatch 2018).

### Amblyiulus georgicus

Taxon classificationAnimaliaJulidaJulidae

Lohmander, 1932

EF9858D7-FB69-56D4-9F44-AD5CF026BDF4

**Figure 1.**
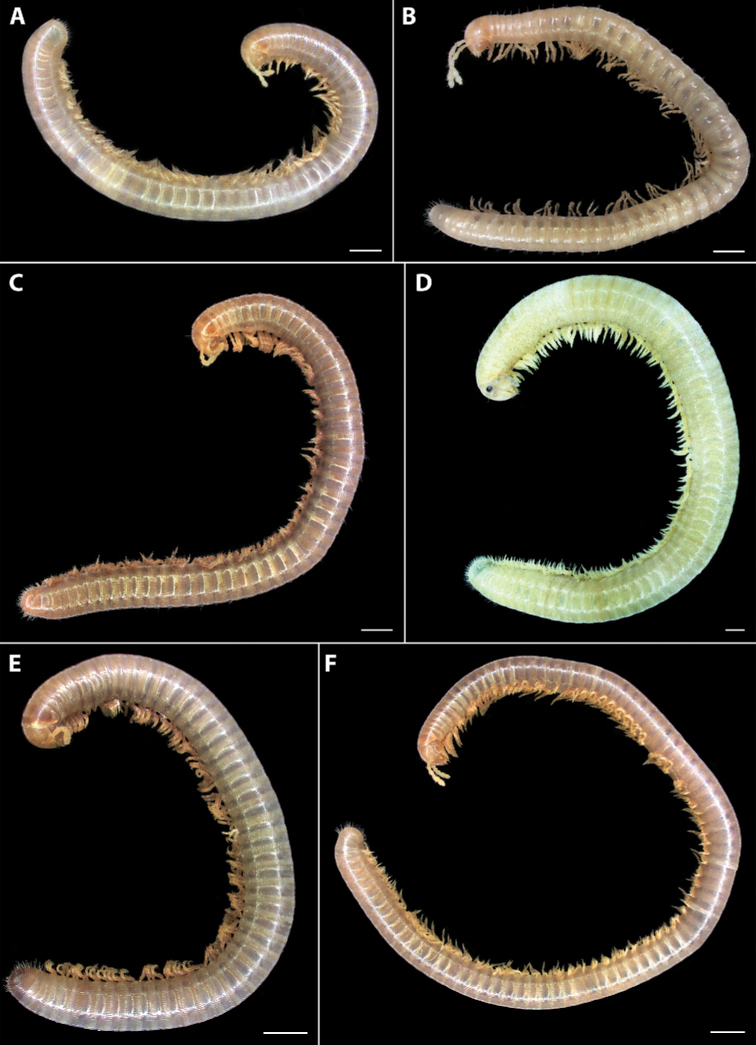
Habitus of *Amblyiulus* and *Syrioiulus* species, males, lateral views **A***A. georgicus* Lohmander, 1932 from Shnokh, Armenia (ZMUM) **B***A. hirtus* sp. nov., paratype from Bash-Layski, Azerbaijan (ZMUM) **C***S. adsharicus* (Lohmander, 1936) from Adigeni, Georgia (ZMUM) **D***S. continentalis* (Attems, 1903) from Istisu, Azerbaijan (ZMUM) **E***S. taliscius* (Attems, 1927) from Avrora, Azerbaijan (ZMUM) **F***S. armeniacus* sp. nov., paratype from Shikahoh, Armenia (ZMUM). Scale bars: 1.0 mm.

**Figure 2.**
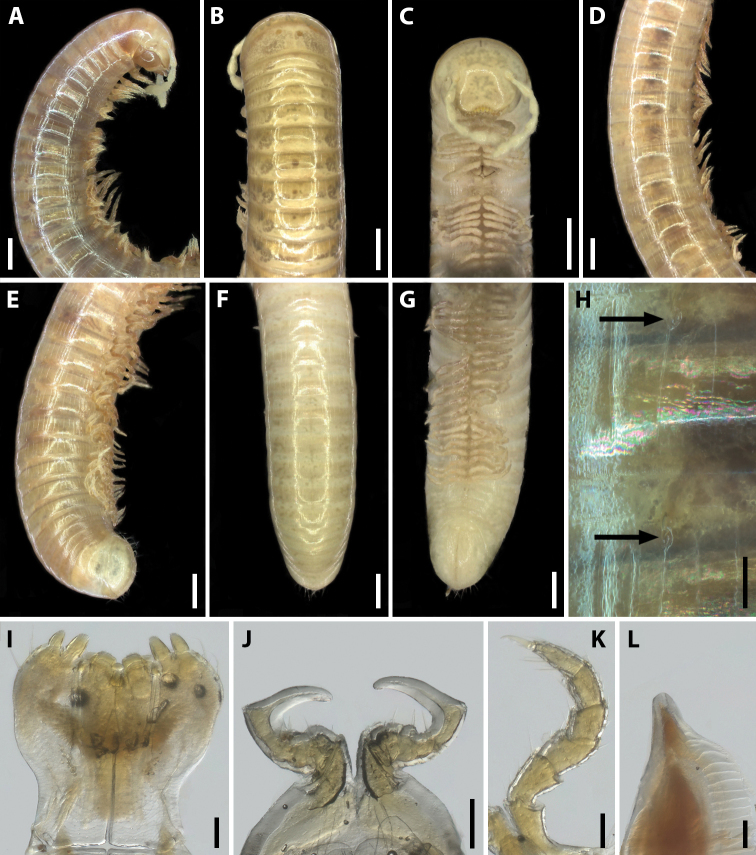
*Amblyiulus georgicus* Lohmander, 1932, ♂ from Shnokh, Armenia (ZMUM) **A–C** anterior part of body, lateral, dorsal and ventral views, respectively **D** midbody part, lateral view **E–G** posterior part of body, lateral, dorsal and ventral views, respectively **H** ozopores on midbody rings, lateral view **I** gnathochilarium, ventral view **J** leg pair 1, caudal view **K** leg 2, caudal view **L** ventral edge of pleurotergum 7, lateral view. Scale bars: 0.5 mm (**A–G**); 0.1 mm (**H–L**).

**Figure 3.**
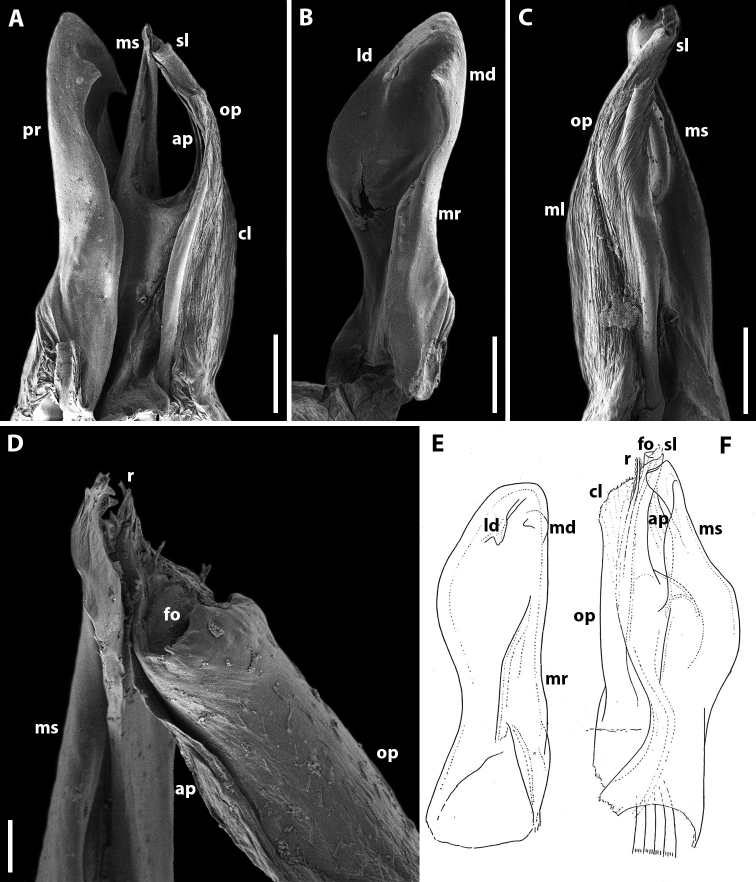
*Amblyiulus georgicus* Lohmander, 1932, ♂ from Shnokh, Armenia (ZMUM) (**A–D**) or holotype ♂, after Lohmander (1932) (**E, F**). **A** gonopod, mesal view **B** promere, caudal view **C** posterior gonopod, caudal view **D** end of solenomere, mesal view **E** promere, caudal view **F** posterior gonopod, lateral view. Abbreviations: **ap** anterior process **cl** caudomesal lamella **fo** fovea **ld** lateral denticle **md** mesal denticle **mr** mesal ridge **ms** mesomeral process **op** opisthomere **pr** promere **r** rod **sl** solenomere. Scale bars: 0.1 mm (**A–C**); 0.01 mm (**D**); not to scale (**E, F**).

Figs 1A , 2 , 3 , 15A , 16

Amblyiulus georgicus Lohmander, 1932a: 180–182, figs 10–12 (D).
Amblyiulus georgicus —Lohmander 1936: 170 (M); Kobakhidze 1965: 395 (M); Lokšina and Golovatch 1979: 385 (M).
Syrioiulus georgicus —Mauriès 1984: 43 (M); Kokhia and Golovatch 2020: 207 (M).

#### Material examined.

**Armenia**: 3 ♂♂, 5 ♀♀, 2 juv. (ZMUM), SW of Shnokh halfway between Alaverdi and Bagratashen, ca. 1500 m a.s.l., *Carpinus* forest, litter, 24.V.1987; 2 ♀♀ (ZMUM), Odzun W of Alaverdi, 1500–1550 m a.s.l., *Quercus*, *Fagus*, *Carpinus*, etc. forest, litter and under stones with ants, 23–24.V.1987; all leg. S. Golovatch, K. Eskov.

#### Diagnosis.

Differs from *A. hirtus* sp. nov., apparently the most similar and geographically the closest congener known to date, by the following combination of somatic and gonopodal characteristics. Head without frontal setae; collum and metazonae of body rings without setae. Male mandibular stipites expanded. Promere wide, with two apical denticles. Solenomere with a membranous lobe notched apically. Rod of opisthomere relatively short. See also Key below.

#### Redescription.

Length of adults 27–30 mm (♂♂) or 28–31 mm (♀♀), width 1.6–1.7 mm (♂♂) or 1.7–1.9 mm (♀♀). Number of body rings in adults, 65–67+2+T (♂♂) or 67–69+2+T (♀♀). Body subcylindrical (typical of Julidae), metazonae brownish grey, prozonae yellowish grey (Fig. 1A). Head, a few postcollum rings and telson lighter than other body rings. Collum more vividly red-brown. Antennae, mouthparts, and legs yellow (Fig. 2A–C). Eyes absent. Metazonae with weak and irregular striations, 14–16 striae between dorsal axial line and ozopore (Fig. 2D). Ozopores relatively large, with a stria in front and lying behind suture without touching it (Fig. 2H).

Antennae relatively long, in situ reaching segment 4. Head without frontal setae, but with 8+8–9+9 labral and 2+2 supralabral setae (Fig. 2A–C). Gnathochilarium with four setae on each lamella lingualis, stipites with a group of several short setae in medial part and three long setae at anterolateral margin (Fig. 2I). Collum and metazonae without setae at posterior margin (Figs 1A, 2A–G). Epiproct undeveloped (Fig. 2E, F). Hypoproct subtriangular, with several long setae (Fig. 2G). Telson and anal valves sparsely setose, setae being long (Fig. 2E–G).

**Male.** Mandibular stipites expanded, slightly swollen in distal part (Fig. 2A). Leg pair 1 small, unciform, with a group of setae on each coxa and at base of telopodite; telopodite relatively long (Fig. 2J). Leg pair 2 with pads on postfemur and tibia (Fig. 2K). Penes short, bifurcate on top. Ventral edge of male segment 7 with elongated and rounded lamellae bordering the gonopodal aperture (Fig. 2L).

Gonopods (Fig. 3). Promere spoon-shaped, relatively wide, constricted in basal third; mesal ridge in apical part forming a small mesal denticle (Fig. 3B, E). Lateral denticle large, well-developed. Mesomeral process simple, flattened, ribbon-shaped, notched on top (Fig. 3A, C, F). Opisthomere tripartite (Fig. 3D). Solenomere long, slightly curved, with caudomesal lamella notched apically; apical part with a fovea and a pointed membranous process. Anterior process of opisthomere appressed to solenomere, with a rounded apex. Anterolateral part of opisthomere with a helicoid rod.

**Female.** First two leg pairs unmodified. Vulva rounded, operculum and bursa equal in height (Fig. 15A). Operculum at apical margin oblique, undivided. Bursa asymmetric, lateral valve higher than mesal one. Each valve with two rows of long setae. Median field of bursa very short, narrow; emargination of median field suboval.

#### Remarks.

This species was described from Borjom (= Borjomi), Georgia (Lohmander 1932a). The above samples represent the first formal records of this species from Armenia. It seems to populate high-montane deciduous forests in the western part of the Caucasus Minor (= Lesser Caucasus) (Fig. 16).

### Amblyiulus hirtus sp. nov.

Taxon classificationAnimaliaJulidaJulidae

072D0180-6F18-5A32-94F0-1F7FD38A08C8

http://zoobank.org/38675EEE-B4FE-4B8B-BDF2-3E77DAADE5BE

**Figure 4.**
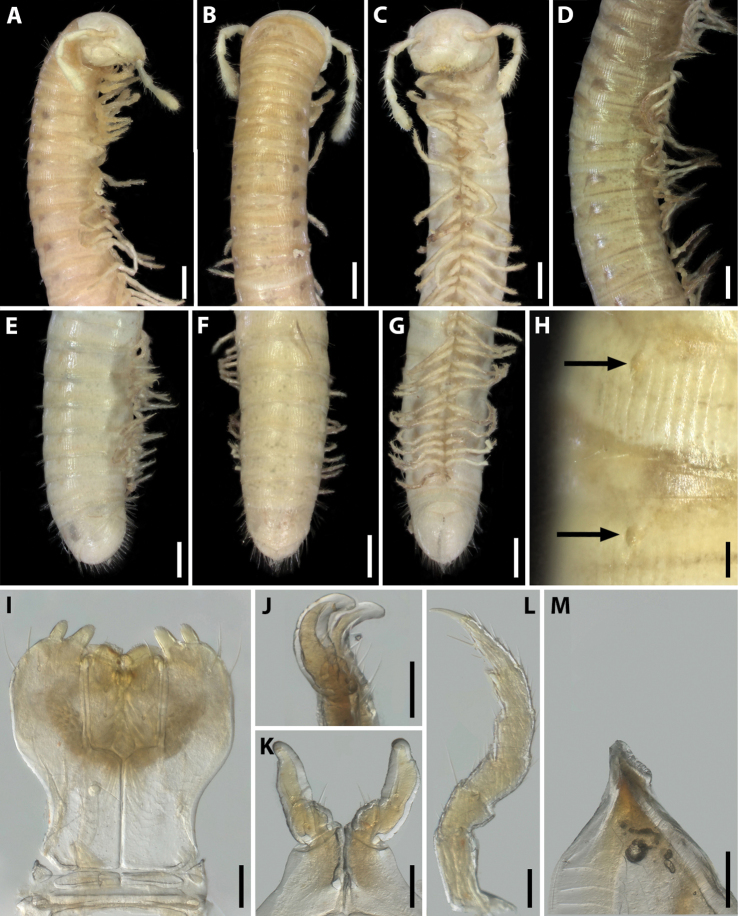
*Amblyiulus hirtus* sp. nov., paratype ♂ from Bash-Layski, Azerbaijan (ZMUM) **A–C** anterior part of body, lateral, dorsal and ventral views, respectively **D** midbody part, lateral view **E–G** posterior part of body, lateral, dorsal and ventral views, respectively **H** ozopores on midbody rings, lateral view **I** gnathochilarium, ventral view **J, K** leg pair 1, lateral and caudal views, respectively **L** leg 2, caudal view **M** ventral edge of pleurotergum 7, lateral view. Scale bars: 0.5 mm (**A–G)** or 0.1 mm (**H–M**).

**Figure 5.**
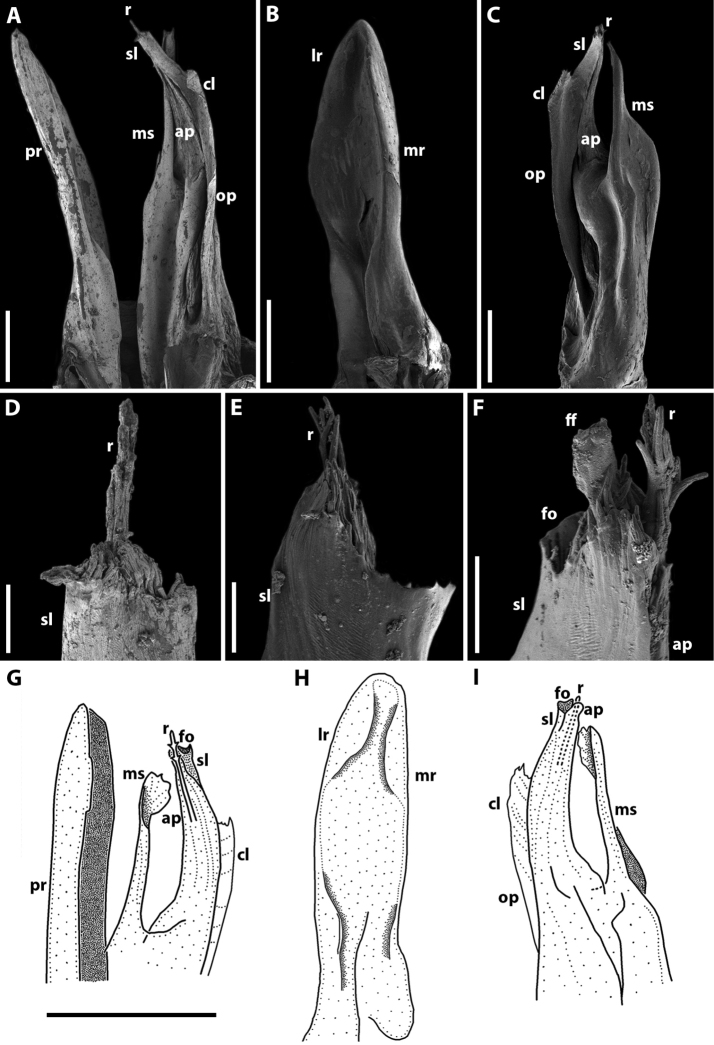
*Amblyiulus hirtus* sp. nov., paratype ♂ from Bash-Layski, Azerbaijan (ZMUM) **A, G** gonopod, mesal and lateral views, respectively **B, H** promere, subcaudal and caudal views, respectively **C, I** posterior gonopod, lateral and mesal views, respectively **D, E** end of solenomere, mesal and submesal views, respectively **F** end of opisthomere, mesocaudal view. Abbreviations: **ap** anterior process **cl** caudomesal lamella **ff** filiform process **fo** fovea **lr** lateral ridge **mr** mesal ridge **ms** mesomeral process **op** opisthomere **pr** promere **r** rod **sl** solenomere. Scale bars: 0.1 mm (**A–C**); 0.01 mm (**D–F**); 0.2 mm (**G–I**).

Figs 1B , 4 , 5 , 15B , 16

#### Material examined.

***Holotype*** ♂ (ZMUM), **Azerbaijan**, NW above Bash-Layski ca. 20 km NNW of Sheki, 1250 m a.s.l., *Fagus*, *Carpinus*, *Acer*, etc. forest, litter, 3.V.1987, leg. S. Golovatch, K. Eskov. ***Paratypes***: 5 ♂♂, 3 ♀♀ (ZMUM), same collection data as holotype.

#### Non-type material.

**Azerbaijan**: 2 ♂♂, 6 ♀♀ (ZMUM), SW of Kuba, 750 m a.s.l., *Fagus*, *Quercus*, *Carpinus*, etc. forest, litter and under bark, 23.IV.1987, leg. S. Golovatch, K. Eskov; **Russia, Dagestan**: 1 ♂, 2 ♀♀ (ZMUM), Kurush, 2550 m a.s.l., S slope, subalpine and alpine meadows, 20.VIII.1990, leg. G. Magomedov.

#### Diagnosis.

Assigned to the genus *Amblyiulus* primarily because of the presence of a rod on the posterior gonopod opisthomere. Differs from *A. georgicus*, perhaps the most similar congener known to date, by the following combination of somatic and gonopodal characters. Head with frontal setae; collum and metazonae of body rings each with a posterior whorl of setae. Promere narrow, with two side ridges. Solenomere apically with small filament-like processes. Rod of opisthomere relatively long.

#### Name.

To emphasise the presence of metazonal setae; adjective.

#### Description.

***Holotype***: length 27 mm, width 1.3 mm, number of body rings 51+2+T. Paratypes: length 25–30 mm, width 1.2–1.4 mm, number of body rings in adults, 45–67+1–3+T (♂♂); or length 27–28 mm, width 1.1–1.3 mm, number of body rings, 46–55+2–3+T (♀♀). Body subcylindrical (typical of Julidae), metazonae and prozonae yellowish grey (Fig. 1B). Head, a few postcollum rings and telson slightly lighter than other body rings. Collum slightly more vividly reddish. Antennae, mouthparts, and legs yellow (Fig. 4A–C). Eyes absent. Metazonae with weak, dense, and regular striations, 21–23 striae per quarter of metazonal surface, i.e., that between dorsal axial line and ozopore (Fig. 4A–G). Ozopores relatively large, with a stria in front, lying behind suture without touching it (Fig. 4H).

Antennae relatively long, in situ reaching ring 4. Head with 1+1 frontal, 8+8–9+9 labral and 2+2 supralabral setae (Fig. 4A–C). Gnathochilarium with three thick setae on each lamella lingualis; stipites without setae in medial part, but with three long setae at anterolateral margin (Fig. 4I). Collum and each following metazona with a whorl of setae at posterior margin (Fig. 4A). Epiproct poorly developed, triangular, with several setae (Fig. 4E, F). Hypoproct subtriangular, covered with long setae (Fig. 4G). Telson and anal valves densely setose, setae being long.

**Male.** Mandibular stipites unmodified (Fig. 4A). Leg pair 1 small, unciform, with a group of setae on coxa and at base of telopodite; telopodites curved anteriad, not anteromesad as in other species of Julidae (Fig. 4J, K). Leg pair 2 with a large pad on tibia and a small one on postfemur (Fig. 4L). Penes short and bifurcate. Ventral edge of male pleurotergum 7 with small subtriangular lamellae bordering the gonopodal aperture (Fig. 4M).

Gonopods (Fig. 5) with anterior and posterior pair equal in height. Promere spoon-shaped, relatively narrow, constricted in basal third; with two ridges: mesal ridge prominent all along; lateral ridge short, located only in apical part of promere (Fig. 5B, H). Mesomeral process simple, flattened, ribbon-shaped, with a small membranous lobe on top (Fig. 5A, C, G, I). Opisthomere tripartite (Fig. 5A, C, E, F). Solenomere long, slightly curved, with a caudomesal lamella notched apically; apical part with a fovea and short filament-like processes (Fig. 5A, C, G, I). Solenomere sometimes with an additional filiform process apically (see Remarks under *Syrioiulus taliscius*). Anterior process notched apically (Fig. 5A, C, E). Rod of solenomere relatively long, consisting of filament-like structures, lateral in position (Fig. 5A, D–G, I).

**Female.** First two leg pairs unmodified. Vulva rounded, operculum higher than bursa (Fig. 15B) and bilobed apically. Bursa asymmetric, lateral valve higher than mesal one. Each valve with two rows of long setae. Median field of bursa very short, narrow; emargination of median field suboval.

#### Remarks.

This species seems to be endemic to the eastern part of the Caucasus Major within both northeastern Azerbaijan and the Republic of Dagestan, Russia (Fig. 16).

It is the presence of a laterally positioned rod that brings both *A. georgicus* and *A. hirtus* sp. nov. particularly close together. However, the rod in these two species is located laterally, whereas that in *A. barroisi* anteromesally (Enghoff 1992: fig. 11; Golovatch 2018: fig. 10C). These differences seem to be quite important, but because those three species share not only the presence of a rod, but also a small, but discernible fovea on top of the solenomere, for the time being it seems best to regard the trio as members of *Amblyiulus*.

### Genus Syrioiulus

Taxon classificationAnimaliaJulidaJulidae

Verhoeff, 1914

04C38549-59AC-5E77-992C-AD4439BDBD6E

#### Type species.

*Dolichoiulus polyzonus* Attems, 1910, by subsequent designation of Jeekel (1971).

#### Diagnosis.

All characters as in *Amblyiulus*, except as follows. Promere usually with two denticles in apical part. Head with or without frontal setae. Eyes present or absent. Opisthomere of posterior gonopod bipartite: a solenomere (with a distinct fovea on top) and an anterior process, vs. tripartite in *Amblyiulus*.

#### Species included.

*Syrioiulus adsharicus* (Lohmander, 1936), *Syrioiulus andreevi* Mauriès, 1984, *Syrioiulus aharonii* (Verhoeff, 1914), *Syrioiulus armeniacus* sp. nov., *Syrioiulus continentalis* (Attems, 1903), *Syrioiulus discolor* (Lohmander, 1932), *Syrioiulus incarnatus* (Lohmander, 1932), *Syrioiulus lohmanderi* Vagalinski, 2020, *Syrioiulus persicus* (Golovatch, 1983), *Syrioiulus polyzonus* (Attems, 1910), *Syrioiulus taliscius* (Attems, 1927), and several others provisionally listed by Vagalinski (2020).

### Syrioiulus adsharicus

Taxon classificationAnimaliaJulidaJulidae

(Lohmander, 1936)

FD1AF8FA-CF67-5EFE-91C4-A30BE3A2CF21

**Figure 6.**
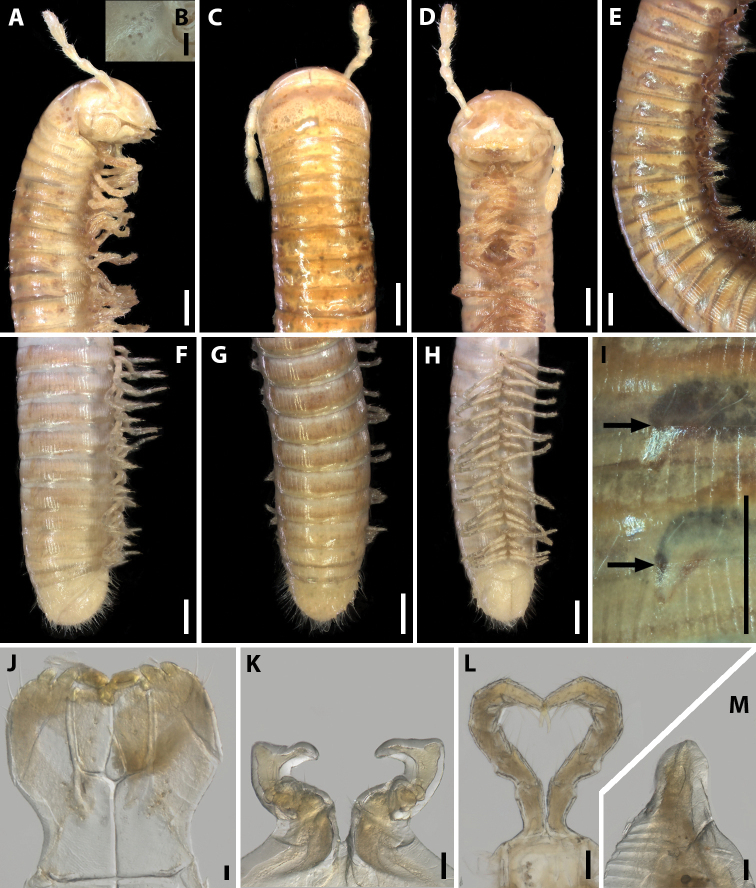
*Syrioiulus adsharicus* (Lohmander, 1936), ♂ from Adigeni, Georgia (ZMUM) **A, C, D** anterior part of body, lateral, dorsal and ventral views, respectively **B** Eye, lateral view **E** midbody part, lateral view **F–H** posterior part of body, lateral, dorsal and ventral views, respectively **I** ozopores on midbody rings, lateral view **J** gnathochilarium, ventral view **K** leg pair 1, caudal view **L** leg pair 2, caudal view **M** ventral edge of pleurotergum 7, lateral view. Scale bars: 0.5 mm (**A, C–I**); 0.1 mm (**B**); 0.05 mm (**J–M**).

**Figure 7.**
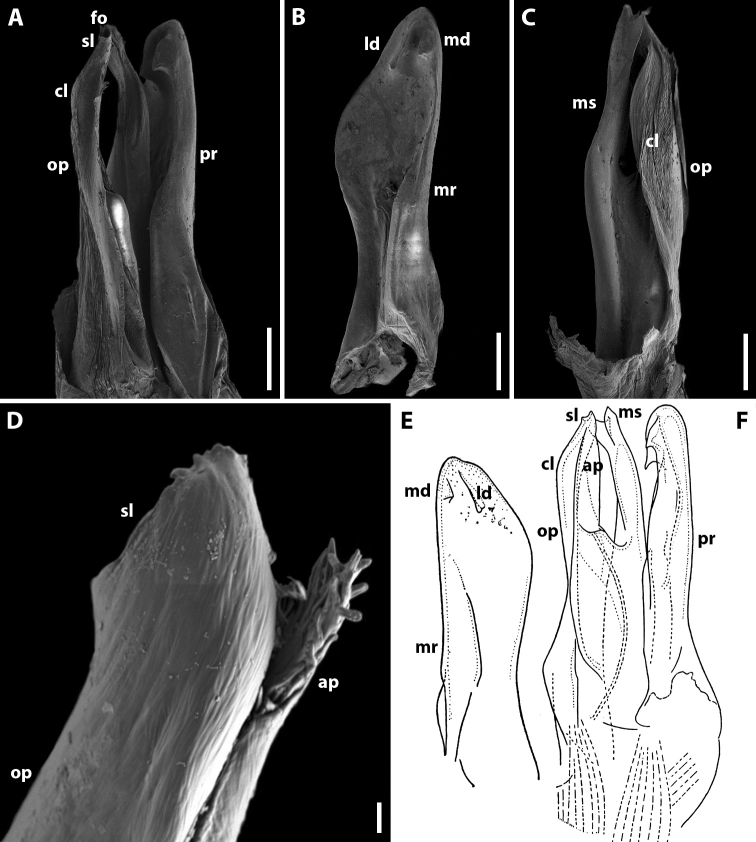
*Syrioiulus adsharicus* (Lohmander, 1936), ♂ from Adigeni, Georgia (ZMUM) (**A–D**) or holotype ♂, after Lohmander (1936) (**E, F**) **A, F** gonopod, mesal view **B, E** promere, caudal view **C** posterior gonopod, lateral view **D** end of solenomere, lateral view. Abbreviations: **ap** anterior process **cl** caudomesal lamella **fo** fovea **ld** lateral denticle **md** mesal denticle **mr** mesal ridge **ms** mesomeral process **op** opisthomere **pr** promere **sl** solenomere. Scale bars: 0.1 mm (**A–C**); 0.03 mm (**D**); not to scale (**E, F**).

Figs 1C , 6 , 7 , 15C , 16

Amblyiulus (Heteropachyiulus) adsharicus Lohmander, 1936: 156–159, figs 131, 132 (D).
Amblyiulus adsharicus —Kobakhidze 1964: 191 (M); Lokšina and Golovatch 1979: 385 (M); Talikadze 1984: 143 (M); Kokhia and Golovatch 2018: 40 (M).
Syrioiulus adsharicus —Vagalinski 2020: 89 (M); Kokhia and Golovatch 2020: 207 (M).

#### Material examined.

**Georgia**: 10 ♂♂, 16 ♀♀, 5 juv. (ZMUM), 15 km W of Adigeni, *Abies*, *Picea*, *Fagus*, *Acer*, etc. forest, 1500–1700 m a.s.l., litter, logs, under stones, 14–15.V.1983, leg. S. Golovatch; 4 ♂♂, 5 ♀♀ (ZMUM), near Adigeni, 9.VI.1977, leg. V. Dolin.

#### Diagnosis.

Differs from all congeners by the following combination of somatic and gonopodal characters. Head with frontal setae. Collum and each metazona of following body rings with a whorl of long setae at caudal margin. Ommatidia present, but only a few ommatidia, all unpigmented and very small. Solenomere with small denticles apically. Anterior process of opisthomere with small filament-like spines apically.

#### Redescription.

Length of adults 17–30 mm (♂♂) or 18–31 mm (♀♀), width 1.2–1.4 mm (♂♂) or 1.3–1.7 mm (♀♀). Number of body rings in adults, 50–63+1–2+T (♂♂) or 52–60+1–2+T (♀♀). Body subcylindrical (typical of Julidae), metazonae brownish grey, prozonae violet grey (Figs 1C, 6A, C). Head, collum and body rings from yellow to greyish yellow. Antennae, mouthparts and first leg pairs yellow, other pairs brown (Fig. 6A, C–H). Eyes present, unpigmented, very small, composed of 3–7 ommatidia, unequal numbers on opposite sides of head (Fig. 6B). Metazonae with weak striations, 17–19 striae per quarter of metazonital surface, i.e., that between dorsal axial line and ozopore (Fig. 6E). Ozopores small, lying behind suture and touching it (Fig. 6I).

Antennae relatively long, in situ reaching segment 4. Head with 1+1 frontal, 11+11–13+13 labral and 2+2 supralabral setae (Fig. 6C, D). Gnathochilarium with four long setae on each lamella lingualis, stipites with a medial group of 7–10 short setae, three long and two short setae at anterolateral margin (Fig. 6J). Collum and each metazona of following rings with a whorl of long setae at posterior margin (Fig. 6A, E, F–H). Epiproct undeveloped (Fig. 6F, G). Hypoproct subtriangular, with long setae (Fig. 6H). Anal valves densely setose, setae being long.

**Male.** Mandibular stipites expanded, slightly swollen (Fig. 6A). Leg pair 1 small, unciform, telopodites curved anteromesad (as in most other Julidae), with a group of setae on each coxa and at base of telopodite (Fig. 6K). Leg pair 2 with pads on postfemur and tibia (Fig. 6L). Penes short, bifurcate. Ventral edge of male pleurotergum 7 with relatively wide and apically rounded lamellae bordering the gonopodal aperture (Fig. 6M).

Gonopods (Fig. 7) with anterior and posterior pair equal in height. Promere spoon-shaped, constricted in basal third; a mesal ridge along basal 2/3 extent; two apical denticles well-developed, mesal one vertical and with a weakly bifurcate apex, lateral one short, wide and rounded apically (Fig. 7B, E). Mesomeral process simple, flattened, ribbon-shaped, bifurcate (Fig. 7C, F). Opisthomere bipartite (Fig. 7D, C). Solenomere long, erect, with small denticles apically, bearing a fovea at apex; caudomesal lamella wide with a notched apical margin (Fig. 7A, C, F). Anterior process apically with small filament-shaped spines (Fig. 7D).

**Female.** First two leg pairs unmodified. Vulva elongated, covered with long setae (Fig. 15C). Operculum relatively low, deeply divided. Bursa asymmetric, lateral valve higher than mesal one. Median field of bursa narrow; emargination of median field narrow and elongated.

#### Remark.

This species was originally described from Batumi, “Bortschacha” (Lohmander 1936). Our new record from near Adigeni is evidence of the species likely to be endemic to the southern part of the Colchidan biogeographic province, all within Georgia (Fig. 16).

### Syrioiulus continentalis

Taxon classificationAnimaliaJulidaJulidae

(Attems, 1903)

80560408-A12F-52E3-9052-2CA9F91DFB0D

**Figure 8.**
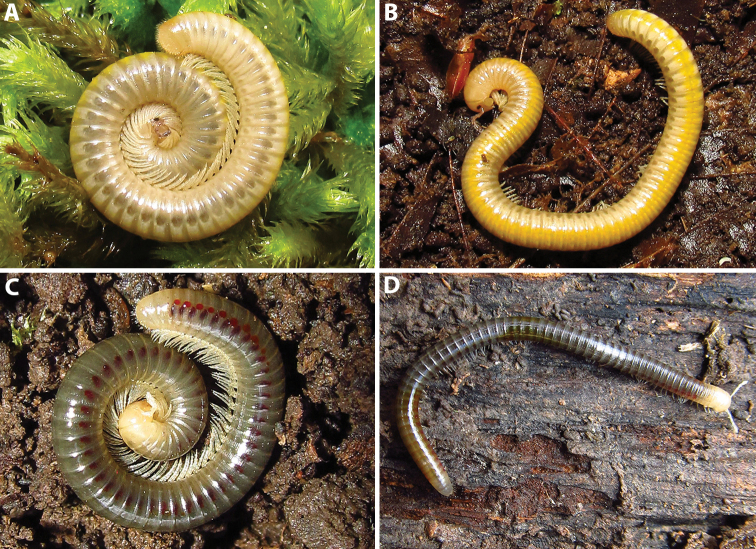
Live colouration of two *Syrioiulus* species from Hyrcan Nature Reserve, Azerbaijan **A, B***S. continentalis* (Attems, 1903) **C, D***S. taliscius* (Attems, 1927). Pictures by D.Ž. Antić, not taken to scale.

**Figure 9.**
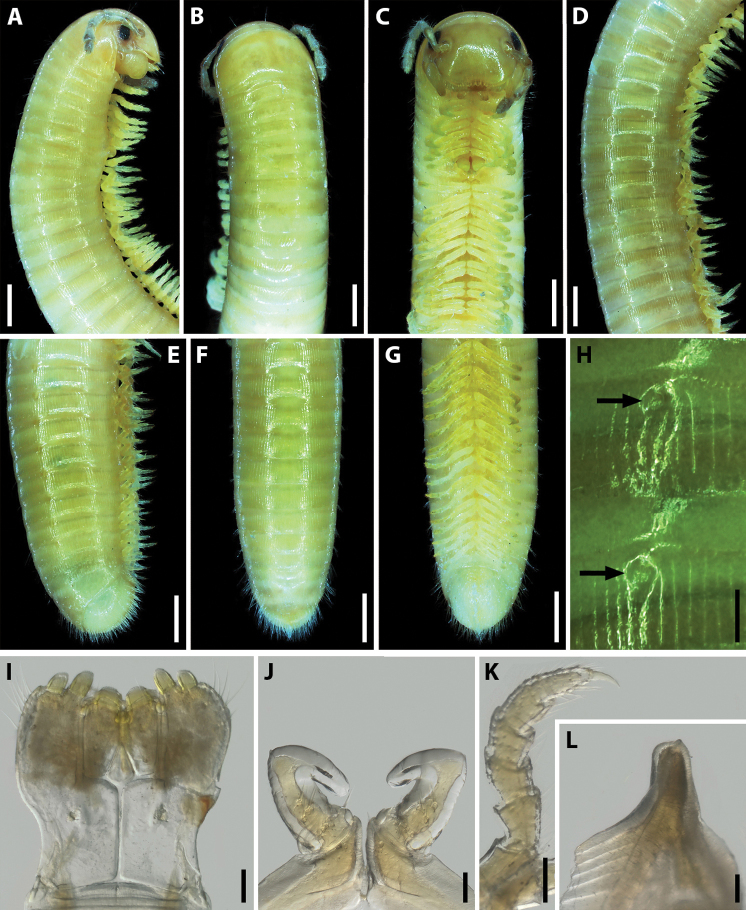
*Syrioiulus continentalis* (Attems, 1903), ♂ from Istisu, Azerbaijan (ZMUM) **A–C** anterior part of body, lateral, dorsal and ventral views, respectively **D** midbody part, lateral view **E–G** posterior part of body, lateral, dorsal and ventral views, respectively **H** ozopores on midbody rings, lateral view **I** gnathochilarium, ventral view **J** leg pair 1, caudal view **K** leg 2, caudal view **L** ventral edge of pleurotergum 7, lateral view. Scale bars: 1.0 mm (**A–G)**; 0.1 mm (**H–L**).

**Figure 10.**
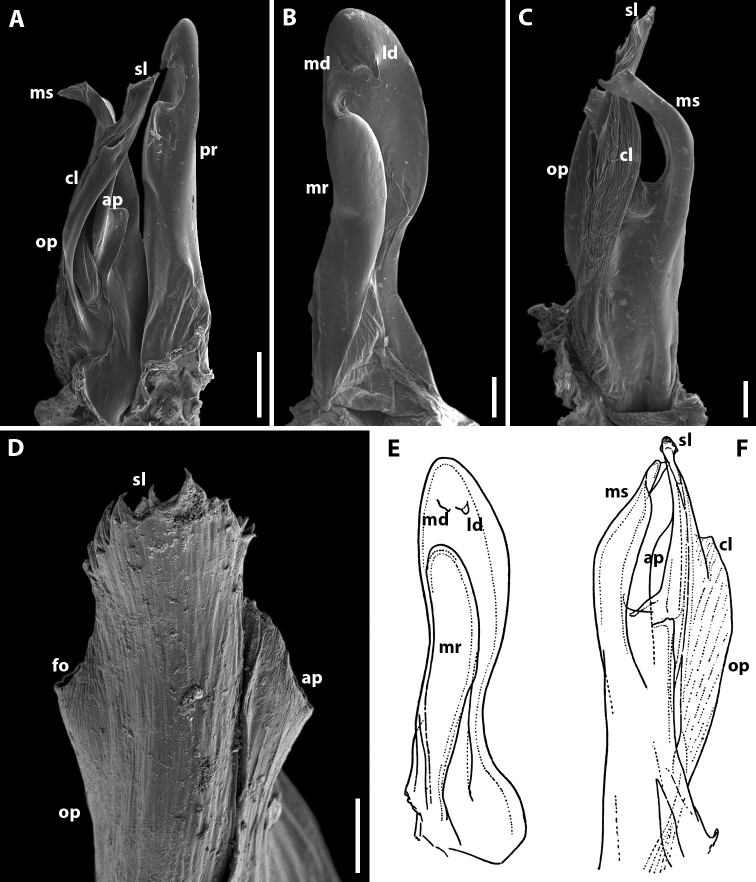
*Syrioiulus continentalis* (Attems, 1903), ♂ from Hyrcan Nature Reserve (**A–C**) (SMNG), from Istisu (**D**) (ZMUM) or after Lohmander (1932b) (**E, F**) **A** gonopod, mesal view **B, E** promere, caudal view **C, F** posterior gonopod, lateral view **D** end of solenomere, lateral view. Abbreviations: **ap** anterior process **cl** caudomesal lamella **fo** fovea **ld** lateral denticle **md** mesal denticle **mr** mesal ridge **ms** mesomeral process **op** opisthomere **pr** promere **sl** solenomere. Scale bars: 0.1 mm (**A–C**); 0.01 mm (**D**); not to scale (**E, F**).

Figs 1D , 8A, B , 9 , 10 , 15D , 16

Pachyiulus (Dolichoiulus) continentalis Attems, 1903: 147, 148, figs 82–84 (D).
Amblyiulus continentalis —Lohmander 1932b: 40, 41, figs 33–35 (D); 1936: 156 (R); Rakhmanov 1971: 1412 (R); 1972: 116 (R); Samedov et al. 1972: 1245; Lokšina and Golovatch 1979: 385 (M); Bababekova 1996: 90 (M).
Syrioiulus continentalis —Mauriès 1982: 441 (M); 1984: 43 (M); Vagalinski 2020: 89 (M).

#### Material examined.

**Azerbaijan**: 3 ♂♂, 1 ♀ (ZMUM), Talysh Mts, Zuvand, Joni, 1500 m a.s.l., 28–29.V.1976, leg. V.G. Dolin; 1 ♂ (ZMUM), Lenkoran, Hyrcan forest, Khan Bulan River near Alexeevka, 22.IV.1985, leg. E.B. Kupriyanova; 7 ♂♂, 5 ♀♀, 2 juv. (ZMUM), Lenkoran, Hyrcan Nature Reserve, litter, 26.I.–4.II.1985, leg. A. Druk; 2 ♂♂, 4 ♀♀ (ZMUM), same locality, 21.IX.1987, leg. S. Zonstein; 2 ♂♂, 2 juv. (IZB); 1 ♂, 1 ♀, 1 juv. (SMNG), Lǝnkǝran rayon, Hyrcan Nature Reserve, Daştatük 1.3 km Xanbulan Reservoir, *Parrotia* forest, diverse bushes, under leaves, 110 m a.s.l., 38.6747°N, 48.7622°E; 1 ♂ (IZB); 1 ♂ (SMNG), same locality, SW of Aşağı Apu, *Quercus* forest, within leaves and rotten wood, 180 m a.s.l., 38.6726°N, 48.7362°E, all leg. F. Walther, H. Reip, D. Antić; 3 ♂♂, 2 ♀♀, 1 juv. (IZB); 5 ♂♂, 3 ♀♀ (SMNG), Lerik rayon, Hyrcan Nature Reserve, road Lǝnkǝran–Lerik at km 32, small side valley, forest of *Parrotia* with some *Quercus*, thick leaf layer, 400 m a.s.l., 38.7638°N, 48.5819°E; 1 juv. (IZB), Astara rayon, Hyrcan Nature Reserve, SW of Zünqülǝş, beginning of a small valley, *Parrotia* and *Alnus* bushes, in leaves, 60 m a.s.l., 38.4493°N, 48.7623°E; 2 ♂♂, 1 juv. (IZB); 2 ♂♂, 1 ♀ (SMNG), same locality, end of small valley, steep slope, *Parrotia*, *Quercus*, *Acer* trees, under leaves and rotten tree trunks, 130 m a.s.l., 38.4480°N, 48.7597°E, all leg. F. Walther, H. Reip, D. Antić; 2 ♂♂ (ZMUM), Azfilial, 100 m a.s.l., 31.V.–1.VI.1996; 1 ♂ (ZMUM), Apo below Bilasar, 350 m a.s.l., 8–9.VI.1996; 2 ♂♂, 1 ♀, 1 juv. (ZMUM), Astara Distr., Istisu ca. 8 km WSW of Astara, *Quercus*, *Acer*, *Carpinus*, etc. forest, 10–30 m a.s.l., litter, under stones and bark, 10.X.1983; 2 ♀♀ (ZMUM), Istisu ca. 8 km SW of Masally, *Quercus*, *Acer*, *Carpinus* etc. forest, 80–140 m a.s.l., under bark and stones, 19–20.X.1983; 2 ♀♀ (ZMUM), Istisu W of Astara, 100 m a.s.l., 2–6.VI.1996, all leg. S. Golovatch.

#### Diagnosis.

Differs from all congeners by the following combination of somatic and gonopodal characters. Head with frontal setae. Collum and each metazona of following body rings with a whorl of long setae at caudal margin. Eyes present. Solenomere with a group of small spines on top. Anterior process of opisthomere subtriangular apically. This species is clearly distinguished in the field from all other millipedes by its characteristic greyish yellow colouration with a yellow stripe dorsally, and its particularly strong odour clearly resembling that of *Pachyiulus krivolutskyi* from the western Caucasus (= Colchis).

#### Redescription.

Length of adults 28–45 mm (♂♂) or 26–46 mm (♀♀), width 2.0–2.3 mm (♂♂) or 2.2–2.7 mm (♀♀). Number of body rings in adults, 46–66+1–2+T (♂♂) or 49–66+1–2+T (♀♀). Body subcylindrical, metazonae from greyish yellow to yellow, prozonae light yellow (Figs 1D, 8A, B); live specimens dorsally with a darker, vivid yellow stripe (Fig. 8B). Head, collum and telson slightly lighter than other body rings (Fig. 8A, B). Antennae grey, mouthparts and legs light yellow (Fig. 9A–G). Eyes present, black, oval, each composed of 19–23 ommatidia (Fig. 9A, C). Striations on metazonae deep, not reaching the caudal margin, 28–32 striae per quarter of metazonal surface, i.e., between dorsal axial line and ozopore (Fig. 9D). Ozopores large, lying behind suture without touching it (Fig. 9H).

Antennae relatively long, in situ reaching segment 3. Head with 1+1 frontal, 8+8–9+9 labral and 2+2–4+4 supralabral setae (Fig. 9A–C). Gnathochilarium with three long setae on each lamella lingualis, groups of several small setae in median part of stipites and six or seven long setae at anterolateral margin (Fig. 9I). Collum and each following metazona with a whorl of long and thick setae at posterior margin (Fig. 9A–F). Epiproct undeveloped (Fig. 9E, F). Hypoproct subtriangular, with long setae (Fig. 9G). Telson and anal valves densely setose, setae being long.

**Male.** Mandibular stipites expanded, with swollen lobes (Fig. 9A). Leg pair 1 small, unciform, telopodites directed anteromesad, with a group of long setae on each coxa; telopodite setose in basal part (Fig. 9J). Leg pair 2 with pads on postfemur and tibia (Fig. 9K). Penes short, bifurcate. Ventral edge of male pleurotergum 7 with narrow elongated lamellae bordering the gonopodal aperture (Fig. 9L).

Gonopods (Fig. 10) with anterior (promere) part higher than posterior (opisthomere) one. Promere spoon-shaped, constricted in basal third; mesal ridge wide along 2/3 extent; with denticles in apical part: mesal denticle small and broadly rounded, lateral one well-expressed and long (Fig. 10B, E). Mesomeral process simple, slightly curved, flattened apically (Fig. 10A, C, F). Opisthomere bipartite (Fig. 10D). Solenomere elongated, with an apical membranous lobe, subtriangular at apex, with a fovea and a group of small spines on top; caudomesal lamella wide (Fig. 10A, C, D, F). Anterior process of opisthomere subtriangular apically (Fig. 10D).

**Female.** First two leg pairs unmodified. Operculum of vulva without setae on caudal surface, apical margin relatively flat (Fig. 15D). Bursa subsymmetrical, lateral valve slightly larger than mesal one. Each valve with two rows of long setae. Median field of bursa narrow; emargination of median field suboval.

#### Remarks.

Probably one of the most common and apparently the largest species of the genus. The unusually strong odour and the chemical composition of the repugnatorial secretion are similar to those of *Pachyiulus krivolutskyi* (Makarov et al., pers. obs.). This species inhabits various deciduous forests in Azerbaijan, also occurring in northern Iran (Lohmander 1932b), endemic to the Hyrcanian biogeographic province (Fig. 16).

### Syrioiulus taliscius

Taxon classificationAnimaliaJulidaJulidae

(Attems, 1927)

366D2F9E-79FA-5C68-BFAE-74A6DE9E6FBC

**Figure 11.**
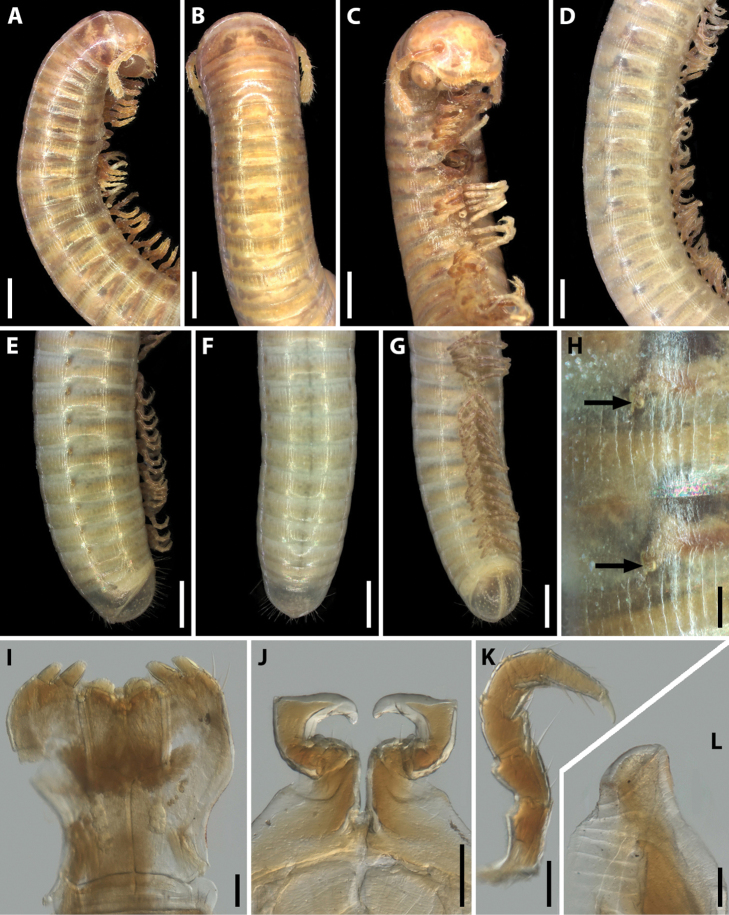
*Syrioiulus taliscius* (Attems, 1927), ♂ from Avrora, Azerbaijan (ZMUM) **A–C** anterior part of body, lateral, dorsal and ventral views, respectively **D** midbody part, lateral view **E–G** posterior part of body, lateral, dorsal and ventral views, respectively **H** ozopores on midbody rings, lateral view **I** gnathochilarium, ventral view **J** leg pair 1, caudal view **K** leg 2, caudal view **L** ventral edge of pleurotergum 7, lateral view. Scale bars: 0.5 mm (**A–G)**; 0.1 mm (**H–L**).

**Figure 12.**
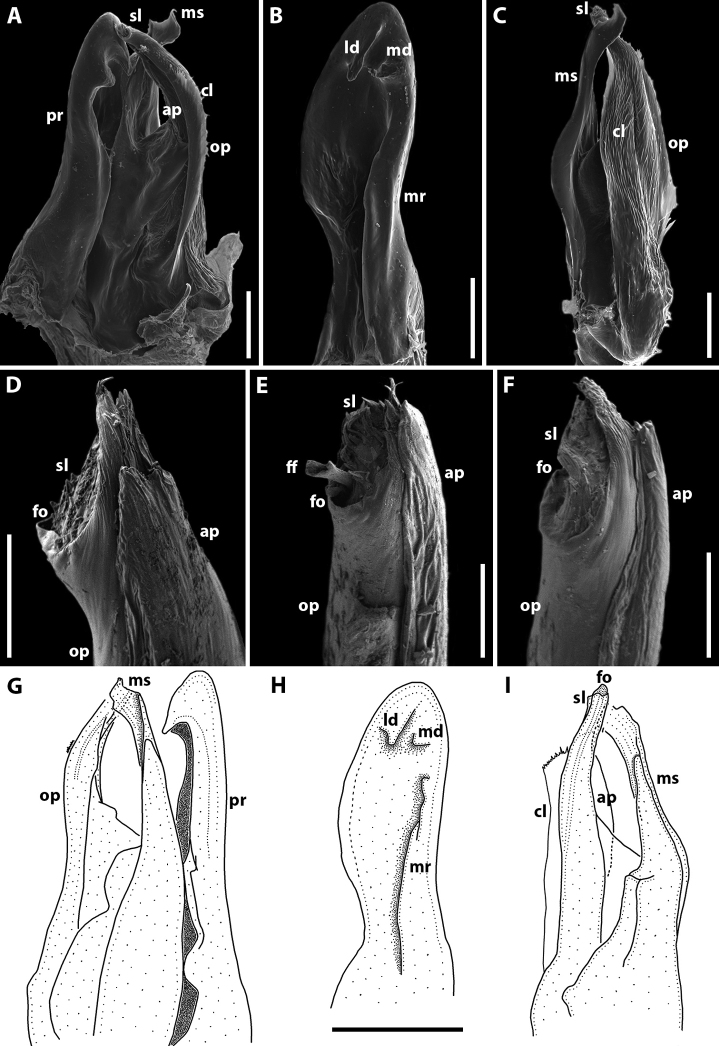
*Syrioiulus taliscius* (Attems, 1927), ♂ from Hyrcan Nature Reserve (**A–C**) (SMNG), from Avrora (**D, G, H**) (ZMUM) or from Apo, Azerbaijan (**E, F**) (ZMUM) **A, G** gonopod, mesal and lateral views, respectively **B, H** promere, caudal view **C, I** posterior gonopod, caudolateral and mesal views, respectively **D–F** end of solenomere, mesal views. Abbreviations: **ap** anterior process **cl** caudomesal lamella **fo** fovea **ld** lateral denticle **md** mesal denticle **mr** mesal ridge **ms** mesomeral process **op** opisthomere **pr** promere **sl** solenomere. Scale bars: 0.1 mm (**A–C**); 0.02 mm (**D–F**); 0.2 mm (**G–I**).

Figs 1E , 8C, D , 11 , 12 , 15E , 16

Amblyiulus taliscius Attems, 1927: 243, 244, figs 336–338 (D).
Amblyiulus taliscius —Lohmander 1932b: 182 (M); 1936: 170 (M); Rakhmanov 1972: 116 (R); Lokšina and Golovatch 1979: 385 (M); Bababekova 1996: 90 (M).
Syrioiulus taliscius —Mauriès 1982: 441 (M); 1984: 43 (M); Vagalinski 2020: 92 (M).

#### Material examined.

**Azerbaijan**: 4 ♂♂, 14 ♀♀, 1 juv. (ZMUM), Talysh, Joni, 1500 m a.s.l., 28–29.V.1976, leg. V.G. Dolin; 2 ♂♂ (ZMUM), Lenkoran, Hyrcan Nature Reserve, Telman, 28.IV.1984; 1 ♂, 1 ♀ (ZMUM), same locality, Gaftoni, 9.V.1984, all leg. H. Aliev; 1 ♀ (ZMUM), Hyrcan forest, Khan Bulan River near Alexeevka, 22.IV.1985, leg. E.B. Kupriyanova; 3 ♂♂, 12 ♀♀ (ZMUM), same locality, Avrora, Moscow-Forest, 50 m a.s.l., 1.VI.1996; 3 ♂♂, 3 ♀♀, 1 juv. (ZMUM), same locality, Apo below Bilasar, 350 m a.s.l., 8–9.VI.1996, all leg. S. Golovatch; 1 ♂ (SMNG), Lǝnkǝran rayon, Siyablı, *Parrotia*, *Zelkova*, *Quercus* coppice, steep slope, 110 m a.s.l., 38.7170°N, 48.7253°E; 1 ♂ (IZB), Lerik rayon, Hyrcan Nature Reserve, road Lǝnkǝran–Lerik at km 32, small side valley, forest of *Parrotia* with some *Quercus*, thick leaf litter, 400 m a.s.l., 38.7638°N, 48.5819°E; 7 ♀♀ (IZB), 7 ♀♀ (SMNG), Astara rayon, Hyrcan Nature Reserve, SW of Zünqüləş, end of small valley, steep slope, *Parrotia*, *Quercus*, *Acer* trees, under leaves and rotten tree trunks, 130 m a.s.l., 38.4480°N, 48.7597°E; 2 ♂♂, 1 ♀, 1 juv. (IZB); 2 ♂♂, 2 ♀♀, 1 juv. (SMNG), Lǝnkǝran rayon, Hyrcan Nature Reserve, Daştatük 1.3 km Xanbulan Reservoir, *Parrotia* forest, divers bushes, under leaves, 110 m a.s.l., 38.6747°N, 48.7622°E; 3 ♂♂, 2 ♀♀, 1 juv. (IZB); 5 ♂♂ (1 SEM), 3 ♀♀ (1 SEM), 1 juv. (SMNG) Lǝnkǝran rayon, Hyrcan Nature Reserve, SW of Aşağı Apu, *Quercus* forest, within leaves and rotten wood, 180 m a.s.l., 38.6726°N, 48.7362°E, all leg. F. Walther, H. Reip, D. Antić; 1 ♂ without gonopods (ZMUM), Shemakha Distr., farm Guseinzade, foothills, *Vitis*, summer 1982, leg. A. Ismailov; 1 ♀ (ZMUM), Zakataly Distr., Geyam, cornfield, 17.IV.1986, leg.?; 7 ♂♂, 5 ♀♀ (ZMUM), Baku, City parks, 18–21.V.1981; 1 ♂ (ZMUM), Belokani near Zakatali, 600 m a.s.l., village garden, 24.V.1981; 1 ♀ (ZMUM), above Akhsu 120 km W Baku, 900 m a.s.l., *Quercus* shrub, 22.V.1981, all leg. S. Golovatch and J. Martens; 1 ♀ (ZMUM), ca. 14 km W of Ismailly, Galyhjakh, under bark, 1.V.1987; 1 ♀ (ZMUM), Altyagach, 1050–1100 m a.s.l., *Quercus*, *Fagus*, *Carpinus*, etc. forest, litter, 20 and 26.IV.1987, all leg. S.Golovatch and K. Eskov; 3 ♂♂, 5 ♀♀, 1 juv. (IZB); 3 ♂♂, 5 ♀♀ (SMNG), İsmayıllı rayon, Topçu 7.8 km towards Vəndam, flat area with old *Fagus* forest, under leaves and dead wood, 630 m a.s.l., 40.9193°N, 48.0027°E; 1 ♂ (SMNG), İsmayıllı rayon, Xanəgah 2 rkm towards İsmayıllı, flat area with old *Fagus* forest, with channels, under leaves, 650 m a.s.l., 40.8233°N, 48.1518°E; 1 ♀ (SMNG), İsmayıllı rayon, S of Zərgəran, slope with *Corylus*, *Clematis* and some *Prunus* trees, stone heaps overgrown by moss, mainly in thick leaf litter and under stones, 880 m a.s.l., 40.7310°N, 48.3680°E, all leg. F. Walther, H. Reip, D. Antić.

#### Diagnosis.

Differs from all congeners by the following combination of somatic and gonopodal characters. Head without frontal setae. Collum and metazonae of following body rings without setae. Eyes absent. Solenomere in apical part with a group of small spines. Anterior process of opisthomere subtriangular apically.

#### Redescription.

Length of adults 26–33 mm (♂♂) or 26–34 mm (♀♀), width 1.2–1.3 mm (♂♂) or 1.2–1.4 mm (♀♀). Number of body rings in adults, 50–65+1–2+T (♂♂) or 49–70+1–2+T (♀♀). Body subcylindrical (typical of Julidae), live specimens with brownish grey pro- and metazonae (Fig. 8C, D), after storage in alcohol prozonae grey, metazonae yellow (Fig. 1E). Head, collum, a few postcollum rings, last body rings, telson and anal valves yellow (Figs 1E, 8C, D). Antennae, mouthparts, and legs light yellow (Fig. 11A–G). Eyes absent. Striations of metazonae deep, not reaching the caudal margin, 23–25 striae per quarter of metazonital surface, i.e., that between dorsal axial line and ozopore (Fig. 11D). Ozopores small, lying behind suture without touching it (Fig. 11H).

Antennae relatively long, in situ reaching segment 3. Head without frontal setae, 9+9–12+12 labral and 2+2 supralabral setae (Fig. 11A–C). Collum and metazonae without setae (Fig. 11A–G). Gnathochilarium with 3–4 long setae on each lamella lingualis, stipites with a medial curved row of 4–5 thick setae and three long setae at anterolateral margin (Fig. 11I). Epiproct undeveloped (Fig. 11E, F). Hypoproct rounded, with several setae (Fig. 11G). Telson covered with long setae, anal valves densely setose.

**Male.** Mandibular stipites unmodified (Fig. 11A). Leg pair 1 small, unciform, telopodites directed anteromesad (as in most Julidae), with long setae on each coxa and in basal part of telopodite (Fig. 11J). Leg pair 2 with pads on postfemur and tibia (Fig. 11K). Penes short, bifurcate. Ventral edge of male pleurotergum 7 with wide curved lamellae bordering the gonopodal aperture (Fig. 11L).

Gonopods (Fig. 12) with anterior and posterior parts both equal in height. Promere spoon-shaped, constricted in basal third; mesal ridge relatively narrow along 2/3 extent; with denticles in apical part: mesal denticle small and broadly rounded, lateral one well-expressed and long (Fig. 12B, H). Mesomeral process simple, slightly curved, with a wide subquadrate lamella apically (Fig. 12A, C, G, I). Opisthomere bipartite. Solenomere elongated, with a caudomesal, notched, membranous lobe, in apical part with a fovea and a group of small spines (Fig. 12A, C–F). Fovea may be equipped with a filiform process (Fig. 12E). Anterior process subtriangular apically.

**Female.** First two leg pairs unmodified. Operculum of vulva without setae on caudal surface, apical margin poorly divided (Fig. 15E). Bursa mostly symmetric, lateral valve slightly larger than mesal one. Each valve with two rows of long setae. Median field of bursa narrow; emargination of median field suboval.

#### Remarks.

This species was described from the Talysh Mts, Lenkoran, Azerbaijan (Attems 1927). In the Caucasus, this is probably one of the most common and widespread congeners. Like *S. continentalis*, it inhabits various deciduous forests, but it can only be considered as subendemic to the Hyrcanian biogeographic province (Fig. 16).

### Syrioiulus armeniacus sp. nov.

Taxon classificationAnimaliaJulidaJulidae

74F6B9A6-23CB-5EFB-B4DE-8FC15CFD2604

http://zoobank.org/64FB28BD-A6C0-43AD-A61E-647AA4964982

**Figure 13.**
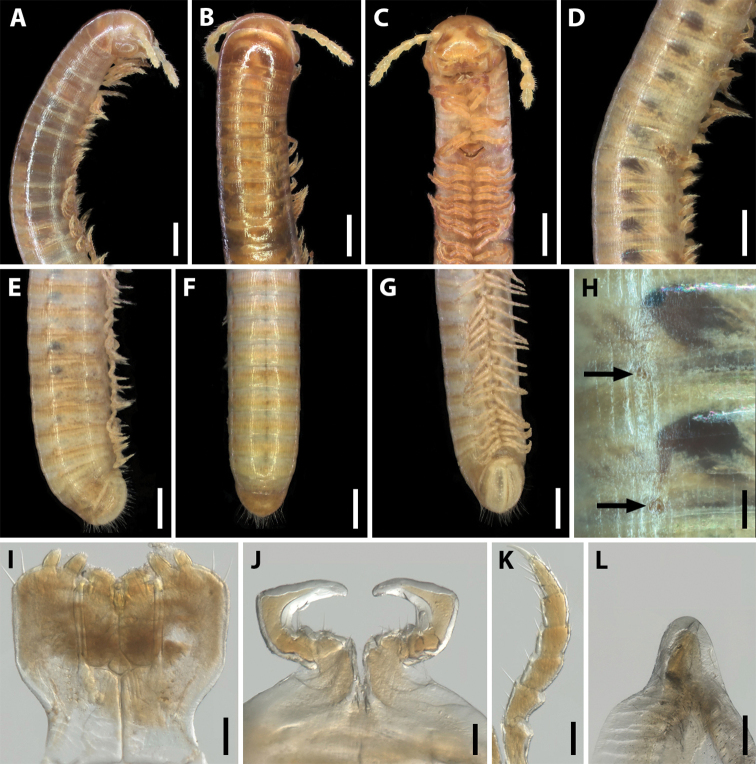
*Syrioiulus armeniacus* sp. nov., paratype ♂ from Shikahoh, Armenia (ZMUM) **A–C** anterior part of body, lateral, dorsal and ventral views, respectively **D** midbody part, lateral view **E–G** posterior part of body, lateral, dorsal and ventral views, respectively **H** ozopores on midbody rings, lateral view **I** gnathochilarium, ventral view **J** leg pair 1, caudal view **K** leg 2, caudal view **L** ventral edge of pleurotergum 7, lateral view. Scale bars: 0.5 mm (**A–G**); 0.1 mm (**H–L**).

**Figure 14.**
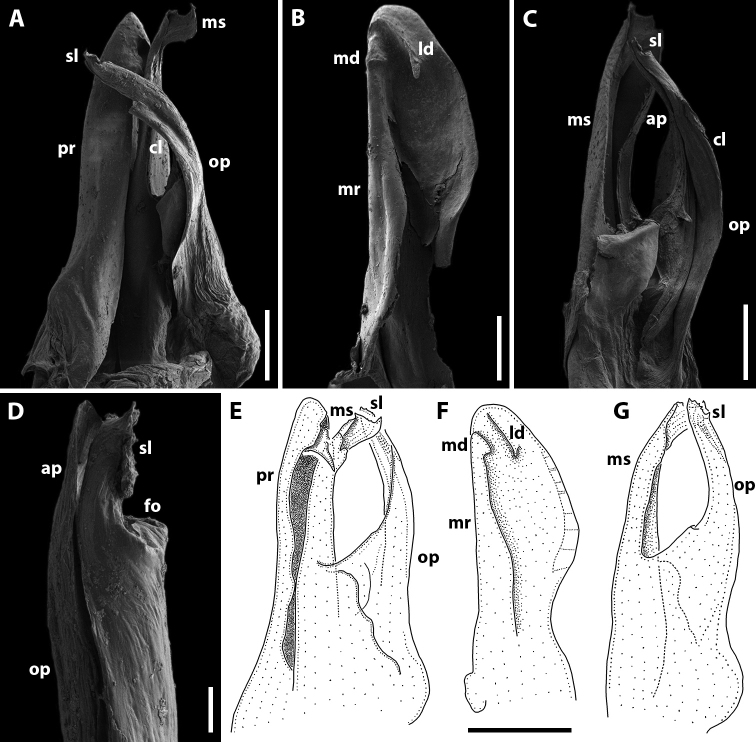
*Syrioiulus armeniacus* sp. nov., paratype ♂ from Shikahoh, Armenia (ZMUM) **A, E** gonopod, mesocaudal and mesal views, respectively **B, E** promere, caudal views **C, G** posterior gonopod, mesal views **D** end of solenomere, mesal view. Abbreviations: **ap** anterior process **cl** caudomesal lamella **fo** fovea **ld** lateral denticle **md** mesal denticle **mr** mesal ridge **ms** mesomeral process **op** opisthomere **pr** promere **sl** solenomere. Scale bars: 0.1 mm (**A–C**); 0.01 mm (**D**); 0.2 mm (**E–G**).

**Figure 15.**
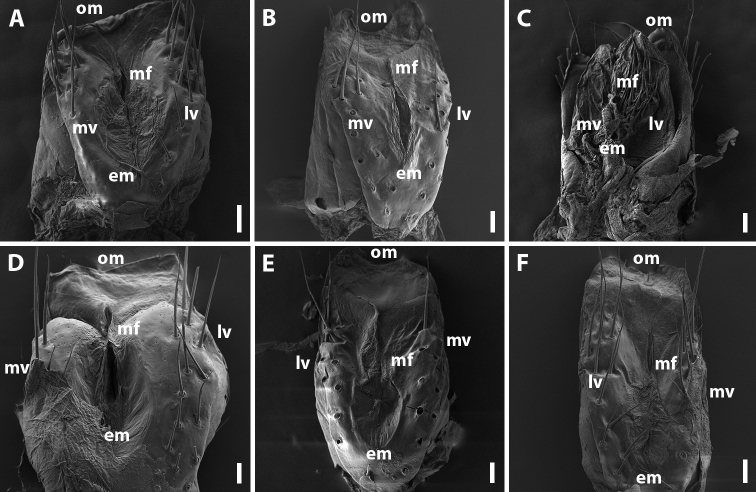
Vulvae of *Amblyiulus* and *Syrioiulus* species from the Caucasus, caudal views **A***A. georgicus* Lohmander, 1932 from Shnokh, Armenia (ZMUM) **B***A. hirtus* sp. nov., paratype from Bash-Layski, Azerbaijan (ZMUM) **C***S. adsharicus* (Lohmander, 1936) from Adigeni, Georgia (ZMUM) **D***S. continentalis* (Attems, 1903) from Istisu, Azerbaijan (ZMUM) **E***S. taliscius* (Attems, 1927) from Avrora, Azerbaijan (ZMUM) **F***S. armeniacus* sp. nov., paratype from Shikahoh, Armenia (ZMUM). Abbreviations: **em** emargination of median field **lv** lateral valve **mf** median field **mv** median valve **op** operculum. Scale bars: 0.03 mm.

**Figure 16.**
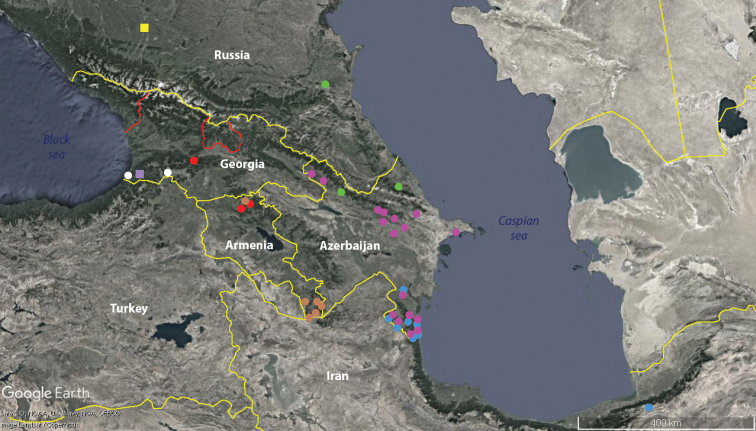
Distributions of *Amblyiulus* and *Syrioiulus* species in the Caucasus: red circle *A. georgicus* Lohmander, 1932; green circle *A. hirtus* sp. nov.; white circle *S. adsharicus* (Lohmander, 1936); blue circle *S. continentalis* (Attems, 1903); pink circle *S. taliscius* (Attems, 1927); brown circle *S. armeniacus* sp. nov.; yellow square Pachyiulini gen. sp. 1; purple square Pachyiulini gen. sp. 2.

Figs 1F , 13 , 14 , 15F , 16

#### Material examined.

***Holotype*** ♂ (ZMUM), **Armenia**, Kafan Distr., Shikahoh Nature Reserve, Shikahoh, 900–950 m a.s.l., *Quercus*, *Fagus*, *Carpinus* forest by spring, litter, logs and under stones, 28.IV.1983, leg. S. Golovatch. ***Paratypes***: 14 ♂♂, 24 ♀♀ (ZMUM), same collection data as holotype.

#### Non-type material.

**Armenia**: 4 ♂♂, 3 ♀♀ (ZMUM), Shikahoh Nature Reserve, Nerkin And, old *Platanus* stand along river, litter, in rotten wood, under stones, 30.IV.1983; 1 ♂, 1 ♀ (ZMUM), near Kajaran, Megri Mt. Ridge, N of Tashtun Pass, 2000 m a.s.l., *Quercus* forest on steep slope, litter, logs, 27.IV.1983; 5 ♂♂, 3 ♀♀, 2 juv. (ZMUM), Megri Distr., SSE of Lichk, Megri River valley, *Quercus* forest, litter, under stones and in rotten wood, 25.IV.1983; 2 ♂♂, 16 ♀♀, 2 juv. (ZMUM), above Kuris, 1500 m a.s.l., *Quercus* and *Carpinus* forest, litter, under bark and stones along spring, 26.IV.1983; 12 ♂♂, 11 ♀♀, 1 juv. (ZMUM), ca. 4 km NNW of Megri, Legvaz, *Juglans* and *Quercus* shrub with *Paliurus* and *Rosa*, litter and under stones, 1000 m a.s.l., 24–25.IV.1983; 5 ♂♂, 2 ♀♀ (ZMUM), 6 km N of Shvanidzor, sparse *Quercus* forest, 1200–1300 m a.s.l., litter, under stones and bark, 24.IV.1983; 5 ♂♂, 6 ♀♀, 2 juv. (ZMUM), environs of Megri, xeriphytous bare canyon, under stones, sparse *Juniperus* and *Paliurus*, ca. 1000 m a.s.l., 24.IV.1983, all leg. S. Golovatch; 2 ♀♀ (ZMUM), Odzun W of Alaverdi, 1500–1550 m a.s.l., *Quercus*, *Fagus*, *Carpinus* etc. forest, litter and under stones with ants, 23–24.V.1987, leg. S. Golovatch and K. Eskov; 1 ♀ (ZMUM), Nurkus, 7.VII.1985, leg. V.A. Zakharyan.

#### Diagnosis.

This new species belongs to the genus *Syrioiulus* because of the presence of only two apices on the opisthomere. Differs from all regional congeners by the following combination of somatic and gonopodal characters. Head with frontal setae. Collum and metazonae of following body rings without setae. Eyes absent. Solenomere with a pointed process apically. Anterior process rounded on top.

#### Name.

The new species is named after its terra typica; adjective.

#### Description.

Holotype: length 25 mm, width 1.2 mm, number of body rings 50+2+T. Paratypes and non-type material: length 17–33 mm, width 1.2–1.6 mm, number of body rings, 50–68+1–2+T (♂♂); or length 20–29 mm, width 1.2–1.6 mm, number of body rings, 46–55+2–3+T (♀♀). Body subcylindrical, metazonae brownish yellow, prozonae brownish grey (Figs 1F, 13A–G). Head, collum and telson slightly lighter than body rings (Fig. 1F). Antennae, mouthparts, and legs yellow (Fig. 13A, C–E, G). Eyes absent. Metazonae with weakly developed, dense, and regular striations, 20–23 striae per quarter of metazonital surface, i.e., that between dorsal axial line and ozopore (Fig. 13D). Ozopores relatively large, situated between striae in touch with one of them, lying behind suture without touching it (Fig. 13H).

Antennae relatively long, in situ reaching segment 3. Head with 1+1 frontal, 9+9–10+10 labral and 2+2 supralabral setae (Fig. 13A–C). Gnathochilarium with three thick setae on each lamella lingualis; stipites with a group of 6–9 setae in medial part and three long setae in anterolateral part (Fig. 13I). Collum and metazonae without setae (Fig. 13A, B). Epiproct poorly developed, triangular, with several long setae (Fig. 13E, F). Hypoproct subtriangular, covered with long setae (Fig. 13G). Telson and anal valves densely setose.

**Male.** Mandibular stipites modified, slightly swollen in distal part (Fig. 13A). Leg pair 1 small, unciform, telopodites directed anteromesad (typical of Julidae), with a group of setae on coxa and telopodite (Fig. 13J). Leg pair 2 with small pads on postfemur and tibia (Fig. 13K). Penes short, bifurcate. Ventral edge of male pleurotergum 7 with wide curved lamellae bordering the gonopodal aperture (Fig. 13L).

Gonopods (Fig. 14) with anterior and posterior parts both equal in height. Promere spoon-shaped, bowl being relatively wide, constricted in basal third; mesal ridge well-developed all along, with a small mesal denticle in apical part; lateral denticle large, rounded on top (Fig. 14B, F). Mesomeral process simple, flattened, ribbon-shaped, widened apically, with a group of small teeth (Fig. 14A, C, E, G). Opisthomere bipartite. Solenomere long, slightly curved; its apical part with a fovea and a pointed process (Fig. 14A, C, D, G). Anterior process as high as solenomere, rounded at tip (Fig. 14D).

**Female.** First two leg pairs unmodified. Vulva rounded, operculum higher than bursa (Fig. 15F). Operculum slightly divided at apical margin. Bursa asymmetric, lateral valve higher than mesal one. Each valve with two rows of long setae. Median field of bursa narrow; emargination of median field elongated and suboval.

#### Remark.

This species seems to be endemic to the Caucasus Minor within Armenia, but most likely it also occurs in the adjacent parts of eastern Azerbaijan and northwestern Iran (Fig. 16).

### Pachyiulini

Taxon classificationAnimaliaJulidaJulidae

gen. sp. 1

F6BBFDA7-159E-5AE8-A68E-94EFFED49F93

Fig. 16

#### Material examined.

1 ♀ (ZMUM) **Russia**, Stavropol Area, Kochubeevsky Distr., Nevinnomyssk, mouth of Bolshoy Zelenchuk River, floodplain forest, 17.IV.2016, leg. R.B. Zuev, A.V. Aulova.

#### Brief description.

Body grey, head and collum dark yellow, antennae and legs yellow. The following somatic characteristics seem to be the most important: absence of eyes, presence of frontal setae, presence of caudal whorls of setae on metazonae, absence of an epiproct, and setose anal valves.

#### Remark.

Unfortunately, the only female does not allow a closer generic allocation.

### Pachyiulini

Taxon classificationAnimaliaJulidaJulidae

gen. sp. 2

D4B62F70-F7AA-55F2-B522-27A43D18661D

Fig. 16

#### Material examined.

**Georgia**: 1 ♀ (ZMUM), Ajaria, Kintrish Nature Reserve, Zeraboseli, 450–600 m a.s.l., 1–3.VI.1981, leg. S. Golovatch, J. Martens; 1 ♀ (ZMUM), same locality, 800 m a.s.l., *Rhododendron* thicket, litter, 13.X.1981, leg. S. Golovatch; 1 ♀ (ZMUM), same locality, valley of Khekpara River, 12.X.1984, leg. E. Kvavadze.

#### Brief description.

Body greyish yellow. Head, collum, a few postcollum rings and telson slightly lighter than other body rings. Ommatidia and frontal setae absent. Collum and each metazona of following rings with a whorl of long setae at posterior margin. Epiproct undeveloped. Anal valves densely setose.

#### Remarks.

These specimens differ from *Syrioiulus adsharicus* in the absence of frontal setae and ommatidia. The absence of males makes it impossible to definitively identify the above samples. At least the taxonomic significance of frontal setae must not be overestimated, as they may be present or absent even within the same species, e.g., *S. aharonii* (see Golovatch 2018).

Since the two unidentified species may well prove to represent *Amblyiulus* or *Syrioiulus*, we map their records in Figure 16, but omit them from the key below.

### Key to *Amblyiulus* and *Syrioiulus* species known to occur in the Caucasus (based on males)

**Table:** 

1	Opisthomere of gonopod with a rod (Fig. 3D, F, G, I)	(genus *Amblyiulus*) **2**
–	Opisthomere of gonopod without rod	(genus *Syrioiulus*) **3**
2	Head without frontal setae, collum and metazonae of body rings without setae (Fig. 2A–C). Promere relatively wide, with two apical denticles (Fig. 3B, E)	***A. georgicus***
–	Head with 1+1 frontal setae, collum and metazonae of body rings with whorls of setae at caudal margin (Fig. 4A–C). Promere narrow, without apical denticles (Fig. 5B, H)	***A. hirtus* sp. nov.**
3	Eyes present	**4**
–	Eyes absent	**5**
4	Larger: width > 2.0 mm. Eyes well-developed, black, oval, each composed of 19–23 ommatidia (Fig. 9A)	***S. continentalis***
–	Smaller: width < 1.3 mm. Eyes present, but very small and unpigmented, each composed of 3–7 ommatidia (Fig. 6B)	***S. adsharicus***
5	Head without frontal setae (Fig. 11A–C)	***S. taliscius***
–	Head with 1+1 frontal setae (Fig. 13A–C)	***S. armeniacus* sp. nov.**

## Discussion

Two allopatric species of *Amblyiulus*, both likely endemic, are found to populate the Caucasus. *Amblyiulus georgicus* inhabits western and central Transcaucasia, while *A. hirtus* sp. nov. seems to be confined to northeastern Transcaucasia and the eastern Caucasus, i.e., occurring on both macro slopes of the Caucasus Major (Fig. 16).

The genus *Syrioiulus* is more diverse and widespread, but presently it seems to be restricted to Transcaucasia. Thus, *S. adsharicus* has a rather narrow distribution in the southwestern parts of the Colchidan biogeographic province. Two most widespread species, *S. continentalis* and *S. taliscius*, are often sympatric to even syntopic within the Hyrcanian biogeographic province, but the latter species also occurs in the Caucasus Minor and the eastern part of the Caucasus Major. *Syrioiulus armeniacus* sp. nov. inhabits the Caucasus Minor within southern and central Transcaucasia (Fig. 16).

As regards the vertical distributions, most species of *Amblyiulus* and *Syrioiulus* in the Caucasus are confined to montane forests, as are probably all *Syrioiulus* species known from Hyrcania, including the Iranian *S. astrabadensis* (Lohmander, 1932b), *S. discolor* (Lohmander, 1932b), *S. incarnatus* (Lohmander, 1932b), *S. lohmanderi* Vagalinski, 2020, *S. persicus* (Golovatch, 1983) and *S. zarudnyi* (Lohmander, 1932b) (Enghoff and Moravvej 2005; Vagalinski 2020). Within the Republic of Azerbaijan, however, *S. taliscius* inhabits not only lowland to foothill woodlands (50–1100 m a.s.l.), but also drier steppe- or bush-clad slopes, being inclined to synanthropisation as well (parks in Baku City). The same generally applies to both *S. continentalis* and *S. armeniacus* sp. nov., as they also populate xerophytic environments. *Amblyiulus hirtus* sp. nov. has been encountered in mid-montane deciduous forests, as well as subalpine and alpine meadows up to 2550 m a.s.l.

Only two species of *Syrioiulus*, *S. continentalis* and *S. taliscius*, are endemic or subendemic, respectively, to the Hyrcanian biogeographic province within the Republic of Azerbaijan and Iran, while the remaining *Amblyiulus* and *Syrioiulus* spp., however provisionally, are formally strictly endemic to the Caucasus sensu lato, including Hyrcania (Fig. 16). Generally, the problem concerning the origins of the *Amblyiulus* and *Syrioiulus* species could be approached through analysing the distribution areas of both these genera. However, because their species compositions are far from settled, including new congeners and records certainly ahead, their exact ranges cannot be outlined at the moment. Based on the highest species diversity estimates, one of the main centres of *Amblyiulus* and *Syrioiulus* speciation could have lain in the Middle East, whence members of both genera might have reached the Caucasus sensu lato. The roles that both Colchis and, especially, Hyrcania, two major, relictual, meso- to hygrophilous biogeographic provinces of, and refugia in, the region concerned, could have played in the evolution and secondary speciation seem paramount. It is hardly random that most pachyiuline species in the Caucasus are encountered in the Caucasus Minor, the immediate northern peninsular continuation of Asia Minor.

Both unidentified species seem to be endemic to the Caucasus, with Pachyiulini gen. sp. 1 being confined to Ciscaucasia, and Pachyiulini gen. sp. 2 to deciduous forests in southern Colchis, occurring sympatrically with *Syrioiulus adsharicus* (Fig. 16).

The above picture is definitely very far from final, but it agrees well with the biogeography of the Caucasus (e.g., Abdurakhmanov 2017). Given that the Pachyiulini in the faunas of Turkey (Enghoff 2006) and Iran (Vagalinski 2020) are likewise quite poorly known, each amounting to only a handful of species, there can hardly be any doubt that future progress in the study of pachyiulines in the Caucasus region and adjacent countries will reveal numerous novelties. In addition, given the presence in Crimea of a troglobitic species, *Syrioiulus kovali* (Golovatch, 2008), finding cave species of Pachyiulini in the Caucasus could also be expected (Golovatch 2008; Golovatch et al. 2017; Turbanov et al. 2018). The present taxonomy and distributions as outlined above must be clarified and refined through future research, both morphology- and molecular-based.

## Supplementary Material

XML Treatment for Amblyiulus

XML Treatment for Amblyiulus georgicus

XML Treatment for Amblyiulus hirtus

XML Treatment for Syrioiulus

XML Treatment for Syrioiulus adsharicus

XML Treatment for Syrioiulus continentalis

XML Treatment for Syrioiulus taliscius

XML Treatment for Syrioiulus armeniacus

XML Treatment for Pachyiulini

XML Treatment for Pachyiulini
